# Recent advances in exploring transcriptional regulatory landscape of crops

**DOI:** 10.3389/fpls.2024.1421503

**Published:** 2024-06-05

**Authors:** Qiang Huo, Rentao Song, Zeyang Ma

**Affiliations:** State Key Laboratory of Maize Bio-breeding, Frontiers Science Center for Molecular Design Breeding, Joint International Research Laboratory of Crop Molecular Breeding, National Maize Improvement Center, College of Agronomy and Biotechnology, China Agricultural University, Beijing, China

**Keywords:** gene regulatory network, transcription factor, multi-omics, transcriptional regulation, crop improvement

## Abstract

Crop breeding entails developing and selecting plant varieties with improved agronomic traits. Modern molecular techniques, such as genome editing, enable more efficient manipulation of plant phenotype by altering the expression of particular regulatory or functional genes. Hence, it is essential to thoroughly comprehend the transcriptional regulatory mechanisms that underpin these traits. In the multi-omics era, a large amount of omics data has been generated for diverse crop species, including genomics, epigenomics, transcriptomics, proteomics, and single-cell omics. The abundant data resources and the emergence of advanced computational tools offer unprecedented opportunities for obtaining a holistic view and profound understanding of the regulatory processes linked to desirable traits. This review focuses on integrated network approaches that utilize multi-omics data to investigate gene expression regulation. Various types of regulatory networks and their inference methods are discussed, focusing on recent advancements in crop plants. The integration of multi-omics data has been proven to be crucial for the construction of high-confidence regulatory networks. With the refinement of these methodologies, they will significantly enhance crop breeding efforts and contribute to global food security.

## 1 Introduction

Plant development and response to environmental stimuli rely on the precise orchestration of gene expression (Zhong et al., 2023). The rich gene expression patterns are governed by multiple regulatory mechanisms, such as gene transcription, mRNA processing, translation, and protein modifications. While gene expression is fine-tuned at different levels, transcriptional regulation is crucial and serves as the primary determinant of the cellular transcriptome (Zhong et al., 2023).

At the transcriptional level, gene expression is controlled by various factors, including transcription factors (TFs) and other DNA-binding proteins. TFs bind to specific genomic binding sites, known as cis-regulatory elements (CREs), within certain chromatin contexts. They can either activate or repress the expression of downstream target genes (Strader et al., 2022). TFs often act in a combination manner, enabling a limited number of TFs to regulate a larger set of target genes (Brkljacic and Grotewold, 2017). The coordinated action of interacting TFs (protein-protein interactions), the interactions between TFs and the promoter DNA of target genes (protein-DNA interactions), and the regulatory relationships among TFs form complex regulatory networks.

Unraveling the transcriptional regulation landscape in plants is important for improving our understanding of the regulatory principles. It allows us to understand how plants respond to internal signals and external environmental variations at the molecular level and how these changes influence plant growth and development. To implement precise genetic engineering strategies in modern breeding, manipulating key transcriptional regulators or their corresponding CREs through genetic engineering can modulate the expression of a set of functional genes or entire metabolic pathways (Grotewold, 2008). A comprehensive understanding of the regulatory networks can help to predict and mitigate potential unintended outcomes of gene editing, thereby improving the yield, nutritional quality, and resistance to diseases or environmental stresses of crop plants. For example, mutation of a target binding site in the *Ideal Plant Architecture 1* (*IPA1*) promoter for an upstream TF has been reported to be able to overcome the tradeoff between the number of grains per panicle and the number of tillers in rice, leading to an increased yield (Song et al., 2022).

Significant advancements have been made in transcriptional regulation studies over the past two decades. With the advent of high-throughput DNA and protein profiling technologies, there is a growing accumulation of multi-omics data. In parallel, developing advanced computational algorithms has facilitated the integration of large-scale datasets, such as transcriptomics, epigenomics, and proteomics, enabling the reconstruction of complex regulatory networks (Depuydt et al., 2023). We are now capable of constructing more accurate network models, which contribute to a deeper understanding of gene regulation. More recently, the application of single-cell sequencing technologies has revealed the heterogeneity of transcriptional profiles at the cellular level, shedding light on the understanding of the dynamic nature of gene regulation during development and stress responses at an unprecedented resolution (Badia-i-Mompel et al., 2023).

In this review, we briefly summarize the characteristics of commonly used molecular networks. We provide an update on various transcriptional regulatory network inference approaches with multi-omics datasets, highlighting recent advances and limitations of each method. Furthermore, we outline the general downstream analyses for the reconstructed networks. Additionally, we highlight the cutting-edge progress of regulatory network studies in crop plants, with a focus on cereals, such as maize and wheat. Finally, the challenges and future directions in the field are discussed.

## 2 Understanding the transcriptional regulation with network-based approaches

**Figure 1:**
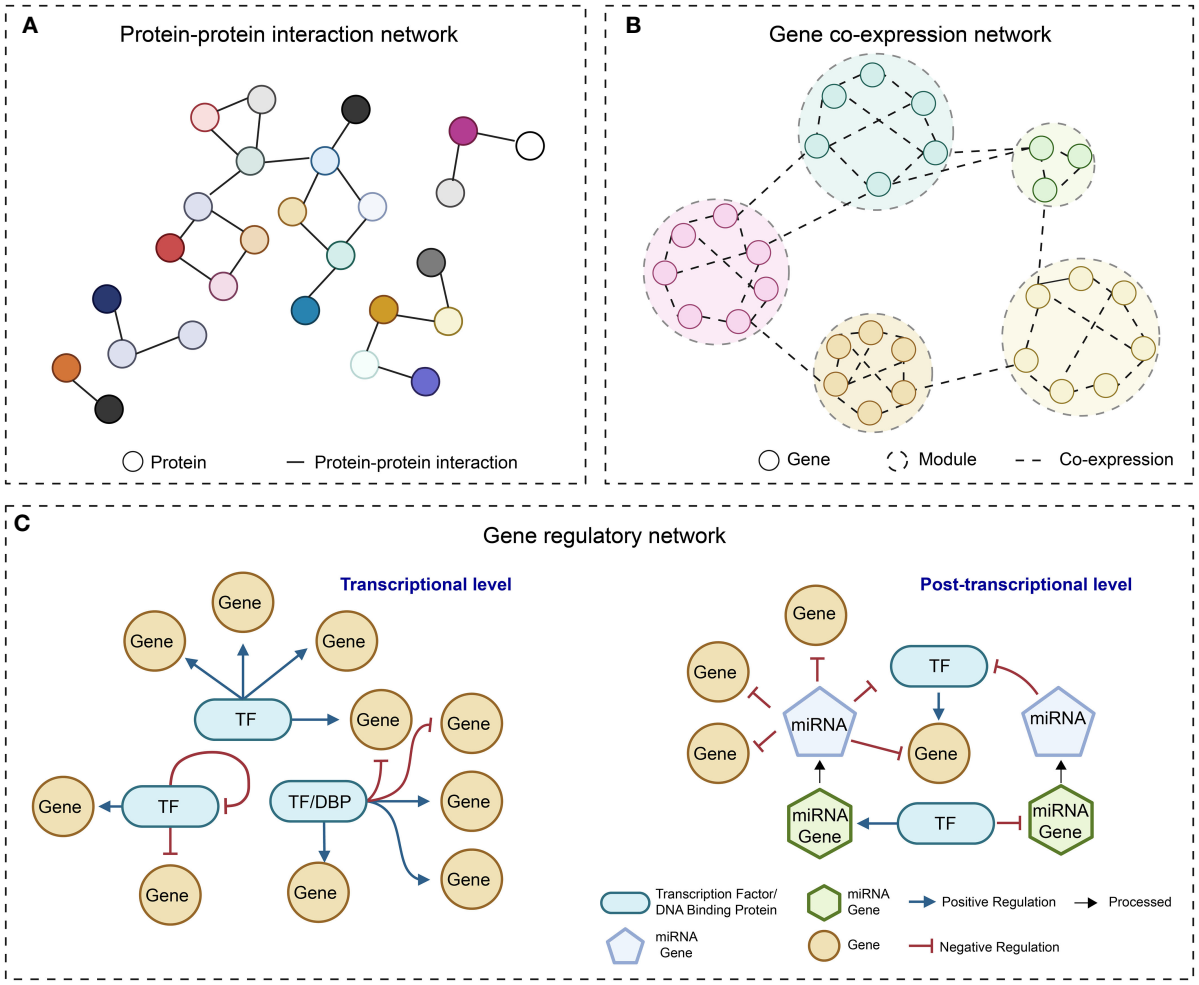
Simple diagrams of different molecular networks. **(A)** protein-protein interaction network; **(B)** Gene co-expression network; **(C)** Gene regulatory network at transcriptional level and post-transcriptional level.

In the post-genomic era, the accumulation of multi-omics data and the rapid progress in developing computational algorithms have empowered us to uncover the complexities of gene function and regulatory programs at a system level. The reconstruction of molecular networks, which are mainly composed of two components (nodes representing biomolecules such as proteins and nucleotides, and edges depicting the interactions between the nodes), is a straightforward approach for visualizing complex interactions and hunting for desirable genes. Most of the currently adopted molecular networks can be classified as protein-protein interaction (PPI) network (Xing et al., 2016), gene co-expression network (GCN) (Rao and Dixon, 2019), and gene regulatory network (GRN) (Van den Broeck et al., 2020) (**Figure 1**).

### 2.1 PPI network: depicting the interactome of proteins

Proteins that share specific biological functions are expected to be interconnected within a PPI network. Therefore, the primary purpose of PPI networks is to unravel the functions of unidentified proteins by using the annotations of known genes. In addition, the network structure information can facilitate addressing biological questions, including the identification of hub proteins, novel pathways, and evolutionary analysis of proteins of interest (von Mering et al., 2002). Noteworthy, the link between two proteins has various implications, such as altering the kinetic properties of enzymes, affecting the substrate binding affinity of effectors, and modifying the regulatory effects of TFs on their downstream target genes (Berggard et al., 2007). Given that transcription regulation depends on both TFs and their associated cofactors, PPI networks offer supplementary insights into transcription regulation (Serebreni and Stark, 2021). This additional layer of information is distinct from the link between TF and regulatory DNA elements.

Currently, only a limited number of experimental-based proteomic networks have been established in plants (Consortium, 2011). Using a yeast-two-hybrid (Y2H) mapping workflow, a comprehensive map of the phytohormone signaling network was constructed, revealing the multifaceted functions of phytohormone proteins in *Arabidopsis* (Altmann et al., 2020). Protein mass spectrometry was also used to identify protein complexes in 13 plant species (McWhite et al., 2020). Recently, Han and colleagues conducted a Y2H screening of 7,623 baits against 21,964 prey proteins in maize, resulting in the identification of 56,243 high-confidence PPIs by vigorous filtering (Han et al., 2023). Moreover, there are computational algorithms have been developed for PPI prediction (Zhang et al., 2016a). A support vector machine (SVM) model has been trained to generate a Protein-Protein Interaction Database for Maize (PPIM), covering ~ 2,700,000 interactions among ~ 14,000 proteins (Zhu et al., 2016). Yang and coworkers have established a comprehensive database (PlaPPISite) for 13 plant interactomes by collecting experimentally validated PPIs and computational predictions (Yang et al., 2020).

Despite these significant advances, well-explored plant PPI maps remain limited. Current data shows that the available PPI datasets cover ~ 12,000 genes in *Arabidopsis*, ~ 40 genes in Soybean, and ~ 300 genes in Rice based on the Biogrid database (accessed on 20 April 2024) (Oughtred et al., 2021). Therefore, it is important to establish a larger quantity of high-quality PPI networks through coordinated efforts by the research communities in the future.

### 2.2 GCN: a useful tool to predict gene function

With the rapid accumulation of transcriptomics data, such as gene expression microarrays and RNA-seq data, GCNs are frequently employed to elucidate the connection between genes and to cluster a large number of genes that exhibit similar expression patterns (Stuart et al., 2003). GCNs represent indirect connections without considering directionality. They are typically generated by a weighted network construction approach followed by hierarchical clustering to identify smaller co-expression modules (Langfelder and Horvath, 2008). While we can use prior knowledge of TF-coding genes to assign the directionality from TFs to their target genes, the directionality between two TFs always remains unknown in the GCN. Despite the lack of causal regulatory links, mounting evidence suggests that GCNs are efficient in predicting the specific biological functions of unknown genes by the “guilt-by-association” principle (Wolfe et al., 2005) and in identifying hub genes that exhibit high connectivity with other genes and may have important regulatory roles (Lin et al., 2019).

To pinpoint regulatory or functional genes involved in specific biological processes, functional modules associated with various pathways or traits are partitioned from large GCNs and annotated by Gene Ontology (GO) terms enrichment analysis. For example, a GCN was constructed and divided into 25 modules in wheat. These modules were annotated and connected to the spatiotemporal progression during wheat endosperm development (Pfeifer et al., 2014); Co-expression modules were also identified for secondary biosynthetic pathways in tea plants (Tai et al., 2018).

As GCNs inherently lack information regarding regulatory relationships among co-expressed genes, it is necessary to combine co-expression analysis with additional complementary data sources, such as cis-regulatory data. Integration approaches can enhance the reliability of GCNs for capturing true biological relevance from network connections. By integration of co-expression data, cis-regulatory elements, and conserved DNA motifs, Vandepoele and coworkers were able to accurately link many unknown genes to specific biological functions, such as the E2F pathway in *Arabidopsis* (Vandepoele et al., 2009).

### 2.3 GRN: a primary approach for investigating regulatory codes

In plants and other organisms, TFs regulate structural genes and TF-coding genes with high context specificity (Badia-i-Mompel et al., 2023). GRN analysis serves as a robust tool for delineating the regulatory relationships between a single or a set of TFs with distinct functions and their downstream target genes in specific cellular and environmental conditions. It has also shown its value in identifying key regulator TFs, regulatory connections between genes and pathways, and in formulating testable functional and regulatory hypotheses (Van den Broeck et al., 2020).

GRNs can be classified into two groups based on their objectives: context-dependent GRNs and comprehensive untargeted GRNs (Depuydt et al., 2023). The majority of GRN studies have been designed to elucidate the network wiring that underlies specific developmental processes or responses to particular environmental conditions. For example, a Bayesian-based network analysis was used to identify multiple genes associated with the *SHOOT MERISTEMLESS* TF gene and to predict their roles in shoot apical meristem development (Scofield et al., 2018). Borrill and colleagues integrated time-series data in wheat and identified several hub genes, including the well-known senescence regulator *NAM-A1*, which regulates the expression of senescence-related genes within the network (Borrill et al., 2019). Zander and coworkers generated a GRN model to predict the cross-talk in the jasmonic acid (JA) signaling pathway and to discover novel components involved in the JA regulatory mechanism (Zander et al., 2020). Furthermore, known and novel candidate TFs were identified associated with water-deficit responses and xylem development plasticity using integrative network analysis in rice (Reynoso et al., 2022).

While context-dependent GRN studies often provide high-resolution information on the specific biological process under investigation, untargeted GRN approaches, despite having lower resolution, are able to capture a broader range of biological processes under various conditions. Untargeted GRNs are typically generated using extensive datasets without focusing on only one specific biological question. Instead, they have been used to establish a database resource or test novel algorithms. For example, Zhou and colleagues collected extensive transcriptome datasets to create coexpression-based GRNs in maize (Zhou et al., 2020). Recently, several resource articles have been published, such as MaizeNetome (Feng et al., 2023), Wheat-RegNet (Tang et al., 2023), and wGRN (Chen et al., 2023b). To introduce more different context-specificities, it is common to incorporate a lot of complementary datasets from various tissues, treatments, and developmental stages. Moreover, the integration of additional omics layers, such as trait-association results, can provide further evidence for the hypotheses drawn from the transcriptome and identify more accurate candidates for the following experimental validation (Kim et al., 2023). Nevertheless, although these GRNs are very large, containing millions of edges, they are not saturated yet.

### 2.4 Inference of gene network in the single-cell era

Single-cell omics technologies, particularly single-cell RNA sequencing (scRNA-seq), provide comprehensive insights into the transcription landscape of diverse plant tissues, surpassing conventional bulk sequencing methods (Rhee et al., 2019). As gene regulation principally takes place in individual cells, inferring regulatory networks based on single-cell data is more effective than using bulk data. It predicts interactions based on expression within the same cells rather than averages (Chen et al., 2019). Moreover, the increased resolution of single-cell omics data allows to capture the cell type- or state-specific GRNs (Aibar et al., 2017).

Current single-cell assays are limited in their ability to detect all transcripts in every cell, often capturing fewer than 5,000 genes per cell. Therefore, specialized tools have been developed to handle this data sparsity (Hao et al., 2024). Common network inference methods designed for scRNA-seq data include SCODE (Matsumoto et al., 2017), PID (Chan et al., 2017), Inferelator (Jackson et al., 2020), and SCENIC/SCENIC+ (Aibar et al., 2017; Van de Sande et al., 2020; Bravo Gonzalez-Blas et al., 2023). These methods vary in their underlying models for linking regulator TFs to target genes. SCENIC first identifies regulatory relationships based on co-expressed genes using GENIE3 (Huynh-Thu et al., 2010) or GRNBoost2 (Moerman et al., 2019), and then refines the connections by considering TF binding motifs on promoter regions. The defined “regulons” consist of co-expressed genes enriched for the CREs to which the regulatory TF binds. Finally, the workflow identifies cells where these regulons are active (Aibar et al., 2017). However, the lack of validated and formatted TF-DNA binding data for most plant species hinders the application of these methods with plant scRNA-seq data.

Single-cell technologies now allow for the quantification of many other modalities, such as scATAC-seq (Buenrostro et al., 2013). GRN methods have been developed to combine the data from multiple modalities (Jiang et al., 2022a; Alanis-Lobato et al., 2024), or alternatively, networks can be constructed separately with each modality and then integrated together (Jansen et al., 2019). Nevertheless, unlike bulk sequencing technologies, which capture a higher number of transcripts, the sparsity inherent in single-cell data may result in biased estimations of gene expression correlations (van Dijk et al., 2018). We expect these challenges to be addressed through enhanced sequencing depths and more sophisticated bioinformatics methodologies to effectively manage data with limited counts (Sekula et al., 2020; Song et al., 2023).

Currently, compared to PPI network and GCN, GRN has emerged as a favored tool for predicting essential regulators and gene expression alterations in response to environmental stimuli and intrinsic signals (Gupta et al., 2022). In some articles, broadly defined GRNs can be formed by the connections between regulatory elements that regulate the transcriptional and translational processes. Such elements, including TFs, splicing factors, and microRNAs, could be incorporated into the modeling of GRNs (Lai et al., 2016; Carthew, 2021) (**Figure 1**). In the following sections, we will use the term “GRN” to refer to the network that abstracts the directed relationships between TFs and their target genes in the context of transcriptional regulation and emphasize studies related to GRNs.

## 3 Reconstruction of transcriptional regulatory networks with multi-omics data

**Figure 2:**
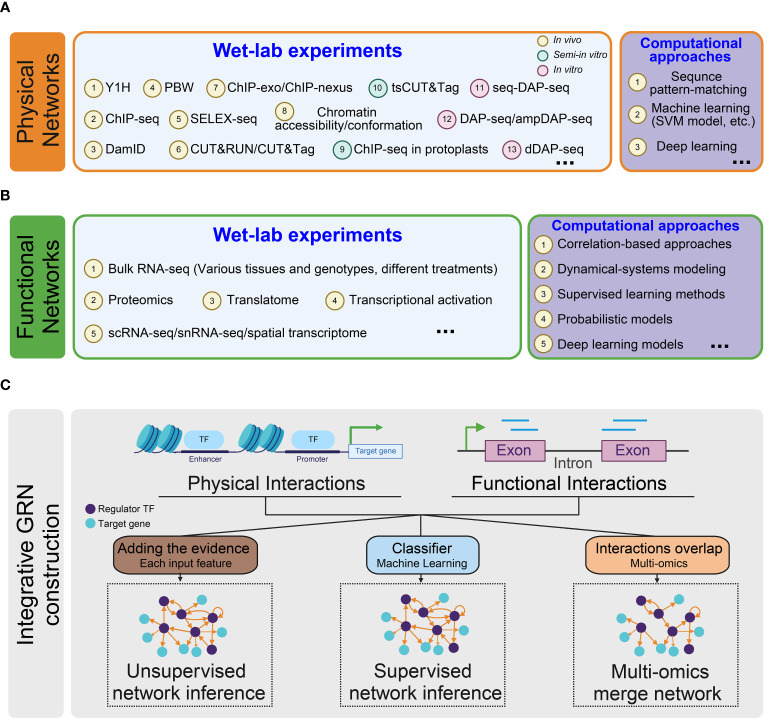
Overview of methodologies for constructing regulatory networks. **(A)** Inference of physical regulatory networks. Two types of methods are employed to construct physical networks: wet-lab experiments (light blue) and computational approaches (purple); **(B)** Inference of functional regulatory networks. Functional networks are inferred using two types of methods: wet-lab experiments (light blue) and computational approaches (purple); **(C)** Inference of integrative regulatory networks. Three types of methods are utilized to infer integrated gene regulatory networks: Unsupervised Network Inference (left): An integrative network is constructed by aggregating evidence from each input feature with equal weighting. Supervised Network Inference (middle): Input features are given to a classification model that predicts the presence or absence of a regulatory interaction for every TF-target pair. Multi-omics integration network (right): This approach identifies regulatory relationships using multi-omics data and merges them into a comprehensive network.

GRNs describe the relationship between target genes and their upstream regulator TFs. Various approaches are used to predict the regulatory edges. These methods can be classified to gene- or TF-centered approaches (Yang et al., 2016) or categorized as experimental techniques and computational inference methods (van der Sande et al., 2023). Here, we adopt a classification based on the source data types of the regulatory link, dividing the networks into three categories: physical, functional, and integrative regulatory networks (**Figure 2**) (Marbach et al., 2012b). The selection of methods for constructing regulatory networks depends on the specific research goals and the availability of relevant data.

### 3.1 Construction of physical GRN

The edges in a physical network represent interactions between a TF and the specific CREs of the target genes it regulates (Schmitz et al., 2022). It is important to note that the physical interaction edges do not imply a functional alteration in gene expression. Instead, they represent a regulation potential that contributes to the complex transcription process.

TFs bind to the genomic TF binding sites (TFBSs) through their DNA binding domains (DBDs). DBDs typically recognize short DNA motifs. Both experimental and computational approaches have been used to identify TFBS and DNA motifs recognized by specific DBDs. Large amount of TFBS and motif datasets have been collected and deposited in public databases, such as TRANSFAC (Matys et al., 2003), CIS-BP (Weirauch et al., 2014), JASPAR (Rauluseviciute et al., 2024), UniPROBE (Hume et al., 2015), PlantPAN (Chow et al., 2024), and ChIP-Hub (Fu et al., 2022).

#### 3.1.1 Identification of TF-DNA interactions using experimental approaches

In addition to the yeast-one-hybrid (Y1H), chromatin immunoprecipitation followed by deep sequencing (ChIP-seq) is a classical technique to identify TF-DNA interactions *in vivo* (Johnson et al., 2007; Gaudinier et al., 2011; Ferraz et al., 2021). New approaches have been developed to address the intrinsic limitations of ChIP-seq, such as its low resolution, low signal-to-noise ratio of detected peaks, and potential enrichment of non-targeted transcription factors (Worsley Hunt and Wasserman, 2014). For example, ChIP-exo and ChIP-nexus can improve the resolution of the detected peaks (He et al., 2015; Rossi et al., 2018). Techniques like CUT&RUN, CUT&Tag, and DamID can eliminate the need for crosslinking and significantly improve the sensitivity of detection (Meers et al., 2019; Alvarez et al., 2020; Tao et al., 2020). However, these methods are still costly and have even more technical complexity, limiting their applications.

Recently, new *in vivo* methods have been developed for large-scale experiments, utilizing tagged TFs transiently expressed in plant protoplasts. These modified ChIP-seq techniques can profile genome-wide TFBSs in an easier and relatively low-cost manner (Lee et al., 2017a; Tu et al., 2020). To further decrease the cost and improve detection sensitivity, Wu and coworkers introduced a transient and simplified CUT&Tag (tsCUT&Tag) method that involves the transient expression of tagged TFs in protoplasts combined with an improved CUT&Tag approach (Wu et al., 2022). This method promises to profile TFBSs more efficiently and cost-effectively across different plant species. Nevertheless, these protoplast-based methods restrict the obtained TF-DNA binding information to the specific tissue source of the protoplasts, such as leaves.

In contrast to *in vivo* methods, *in vitro* approaches eliminate the prerequisite for preparing antibodies specific to TFs or generating transgenic lines containing tagged TFs of interest. Therefore, they can be easily applied in a high-throughput manner (Ferraz et al., 2021). In protein binding microarrays (PBM) and systematic evolution of ligands by exponential enrichment-sequencing (SELEX-seq) methods (Berger and Bulyk, 2006; Smaczniak et al., 2017), TFs or TFs complexes, along with either immobilized DNA oligonucleotides or a random DNA library, are used in these tests. One limitation of PBM is that it may overlook longer TFBSs because it relies on short DNA oligos (10–12 base pairs). Similarly, DNA substrates in SELEX assays are not derived from genomic sequences and cannot be mapped to the genome. To address these limitations, DAP-seq is a recently developed method that utilizes fragmented genomic DNA. In this way, DAP-seq captures more native genomic features, such as DNA methylation and the flanking sequences of core motifs (Bartlett et al., 2017). Nonetheless, DAP-seq method does not fully capture chromatin state information or the cofactors of TFs that can influence TF-DNA binding *in vivo*. To solve these issues, modified methods such as sequential DAP-seq (seq-DAP-seq) and double DAP-seq (dDAP-seq) techniques have been developed (Lai et al., 2020; Li et al., 2023a). In seq-DAP-seq, a sequential purification based on multiple tags was used. Lai and colleagues determined the genome-wide binding of the SEP3 homomeric complex using this method (Lai et al., 2020); dDAP-seq was used to elucidate the DNA binding and specificity of bZIP TF heterodimers and homodimers in *Arabidopsi*s. This study demonstrates that heterodimerization of C/S1 family bZIP TFs expands their DNA binding preferences (Li et al., 2023a). To better reflect *in vivo* TF binding events, the DAP-seq data can be combined with accessible chromatin regions (ACRs) identified by ATAC-seq, DNase-seq, and MNase-seq. The results filtered by tissue- or cell-type-specific ACRs provide more accurate TFBSs by considering *in vivo* chromatin states.

#### 3.1.2 Computational approaches used to predict the TF-DNA interactions

Taking advantage of the extensive experimentally determined TF-DNA interaction data, computational methods have been developed to make *de novo* prediction of TFBS for a given TF. Quantitative models of DNA motifs, such as the Position Weight Matrix (PWM), are required to depict the TF-DNA binding affinity and predicting new DNA binding sites (Jayaram et al., 2016). PWM-based motifs are often built using tools implemented in the HOMER (Heinz et al., 2010) and MEME Suite (Bailey et al., 2015) software collections. These motif discovery algorithms utilize a collection of TFBSs derived from ChIP-Seq, ATAC-seq data, or promoter analyses. Although PWMs provide a good approximation, this conventional model could be further improved by integrating sequence dependencies and DNA shape features (Lai et al., 2019).

Generally, two types of *in silico* approaches can be used to predict the TF binding to the genomic TFBSs: one relies on simple sequence pattern matching, and the other utilizes machine learning algorithms. The pattern matching-based algorithms follow the principle that candidate DNA binding sites possess sequence similarity with known DNA binding motifs of a TF. Several motif search algorithms, such as FIMO, MOODs, and PWMScan, are frequently adopted for this purpose (Grant et al., 2011; Korhonen et al., 2017; Ambrosini et al., 2018). Recent research indicates that pattern-matching-based methodology can be effectively applied across a diverse range of organisms (Puig et al., 2021; Chow et al., 2024). In plant species, PlantRegMap, which now incorporates PlantTFDB V5.0, is a major source for the inferred TF-DNA interactions. It now covers 165 species across the main lineages of green plants (Jin et al., 2017; Tian et al., 2020). However, due to the availability of known DNA motifs, most of the TF-DNA interactions have been identified in *Arabidopsis*.

Machine learning-based approaches establish predictive criteria by learning from documented TF-TFBS data using diverse computational strategies. For instance, Lee and coworkers introduced a SVM model incorporating various features of TFs and TFBSs, achieving approximately 82% prediction accuracy (Lee et al., 2017c). A recent study achieved a remarkable 99% accuracy in model prediction by integrating the chemical properties of TF proteins, along with the structural conformation and bonding capabilities of both TFs and DNA (Khamis et al., 2018). In plants, a SVM model was constructed to identify potential TFBSs for auxin response factor TFs in *Arabidopsis* (Cui et al., 2014). The TSPTFBS (v2.0) employed deep learning to model a total of 389 plant TFs with their binding sequences and achieved better performance than other standard methods (Cheng et al., 2023). Ruengsrichaiya and colleagues developed another machine-learning based predictor (Plant-DTI). This tool leverages a large number of experimental TF-TFBS interactions from plant species with a novel feature construction, resulting in a pronounced high predictive performance compared to other state-of-the-art methods (Ruengsrichaiya et al., 2022).

#### 3.1.3 Connecting TF and target genes with chromatin accessibility and conformation data

Epigenetic modifications and chromatin states are essential factors in regulating gene expression. *In vivo*, most TFs bind to their target CREs within ACRs (Schmitz et al., 2022). Therefore, the identification of ACRs is an important aspect in the study of transcriptional regulation. Optimized genome-wide assays, such as DNase-seq (Boyle et al., 2008), MNase-seq (Mieczkowski et al., 2016), ATAC-seq (Buenrostro et al., 2013), and FAIRE-seq (Simon et al., 2012), have enabled the profiling of chromatin accessibility in numerous species and tissues. Currently, ATAC-seq has emerged as a prominent technique owing to its requirement of a reduced amount of nuclei input and the simplicity of its protocol (Buenrostro et al., 2013). Single-cell ATAC-seq (scATAC-seq) protocols have also been developed and optimized to allow the detection of open chromatin in individual plant cells (Buenrostro et al., 2015; Marand et al., 2021).

In addition to supporting and refining other regulatory networks (Duren et al., 2017), ACR datasets could be directly used in linking TFs to their target genes. This type of network inference pipeline consists of two main steps. Firstly, motif matcher algorithms, provided with TF binding motif data, are used to determine the interactions of TFs with accessible CREs. While scanning TF motifs in ACRs is the routine way, more and more advanced deep learning-based methods are employed to predict the TF binding sites directly from ATAC-seq data (Cazares et al., 2023). Then, these CREs are linked to genes based on a simple distance cutoff or a more refined assignment. These association relationships are combined to obtain “TF-CRE-gene” links and simplified to TF-gene pairs. The GRN inference based on ATAC-seq data can be accomplished with several software packages, such as ATAC2GRN (Pranzatelli et al., 2018), LISA (Qin et al., 2020), SPIDER (Sonawane et al., 2021), and MINI-AC (Manosalva Perez et al., 2024).

Although CREs bound by regulator TFs are often assigned to target genes based on closest genomic proximity, this simplistic approach may miss crucial distal interactions that have regulation effects. Accurately linking CREs to genes can be a challenge task, especially in large genomes that have many distantly located regulatory sequences (Ricci et al., 2019; Joly-Lopez et al., 2020). Chromatin is highly organized to form a three-dimensional (3D) structure. Techniques for measuring chromatin conformation, such as Hi-C and ChIA-PET, were used to capture the long-range chromatin interactions (Lieberman-Aiden et al., 2009; Ouyang et al., 2020). DNA conformation data has been successfully integrated with both ATAC-seq and RNA-seq data to construct GRNs (Jiang et al., 2022b). The characterization of gene regulatory systems based on 3D proximity can be achieved using methods such as DC3 (De-Convolution and Coupled-Clustering) (Zeng et al., 2019).

### 3.2 Inference of functional GRN

A functional regulatory network is characterized by TF-target edges that are supported by changes in the expression patterns of the target genes. These connections, whether direct or indirect, can reflect the functional impact of the regulator’s actions on their targets. From bulk RNA-seq data to single-cell transcriptomics, advanced inference methods have been developed, demonstrating enhanced accuracy and computational efficiency (Marbach et al., 2012a; Pratapa et al., 2020). Several approaches, which utilize time-series data or pseudo-temporal single-cell transcriptomics, were designed to gain more precise insights into the regulatory interactions between genes (Huynh-Thu and Geurts, 2018; Aubin-Frankowski and Vert, 2020).

Several statistical approaches are used for the transcriptome-based gene network analysis. The underlying principles of these methods include correlation, supervised learning, probabilistic models, dynamical-systems modeling, and deep learning (Li et al., 2015; Kim et al., 2023).

#### 3.2.1 Correlation-based approaches

Co-expressed genes are believed to be functionally relevant or co-regulated. A regulatory link between TF and its target may be assumed by the co-expression pattern. The Pearson correlation coefficient (PCC), Spearman’s rank correlation coefficient (SCC), and mutual information (MI) coefficient are popular measures of gene’s co-expression patterns.

PCC is suitable for detecting linear correlations, whereas SCC is more robust to nonlinear relationships. Compared to linear correlation, nonlinear correlation is capable of detecting more complex relationships, which may better reflect the *in vivo* regulatory interactions (Zuin et al., 2022). The MI coefficient is a method based on information theory. It quantifies the interdependence between two variables and can detect nonlinear relationships (Song et al., 2012). However, co-expression analysis is unable to distinguish direct and indirect connections. There may be two ways to solve this issue. One involves the computation of partial correlation coefficients among genes (de la Fuente et al., 2004); the other entails incorporating additional evidence from other data sources, including TF-DNA bindings and ATAC-seq.

#### 3.2.2 Supervised learning methods

Supervised learning methods, such as linear regression, nonlinear regression, and tree-based approaches, are widely used for regulatory network construction.

The linear regression approach first collects expression data for a set of genes as the predictor variables and then regress on the expression levels of designated regulator TFs (response variable). The limitations of regression models include the risk of overfitting due to a large number of predictors (which is a common case in biological systems), and the challenges associated with high-dimensional data. These factors collectively impede the accurate inference of gene regulatory networks (Kim et al., 2023).

In contrast to linear regression, tree-based techniques like random forests have the ability to capture complex non-linear associations among genes (Huynh-Thu et al., 2010). These methods recursively divide the data into smaller subsets based on the predictor variables, creating a tree-like structure of decision rules. Each tree branch represents a distinct combination of predictor values, leading to a predicted value for the target gene at the leaf nodes. Notably, in the DREAM5 challenge (Marbach et al., 2012a), inference tools employing the random forest algorithm achieved the superior overall performance. However, these non-parametric approaches are often less interpretable than linear models. Additionally, they can be computationally intensive, especially when dealing with high-dimensional datasets.

#### 3.2.3 Probabilistic models

Probabilistic models combine principles from probability theory and graph theory to construct networks. These methods capture the dependence between variables, such as transcription factors and their target genes, by modeling the presence and strength of regulatory relationships. Bayesian and Markov are two main types of probabilistic models.

In a Bayesian network, the target gene expression levels are assumed to follow a normal distribution conditioned on the expression levels of TF (Friedman, 2004). Bayesian networks are directed graphs that represent causal relationships between TFs and targets. However, they are unable to reflect feedback regulation relationships, as they do not have loops in the graph structure.

#### 3.2.4 Dynamical-systems modeling

Dynamical systems-based approaches estimate the temporal expression patterns of genes. The regulatory influences of TFs, basal transcription, and inherent stochasticity can be modeled as parameters in differential equations (Hecker et al., 2009). Unlike regression and probabilistic-based approaches, dynamical-systems not only account for the diverse factors that regulate gene expression but also incorporate stochasticity. For example, the observed expression variation among individual cells is biologically meaningful in single-cell RNA-seq data. Dictys method has been developed to utilize the influencing factors through an empirical linear stochastic differential equation (Wang et al., 2023a). It can capture changes in regulatory activity that are not solely dependent on gene expression levels, making it well-suited for studying continuous processes like cell differentiation (Wang et al., 2023a).

#### 3.2.5 Deep learning models

Deep learning models, based on artificial neural networks, offer versatile architectures capable of performing various tasks (Min et al., 2017). Unlike other methods, deep learning models show increasingly improved performance as the size of the training dataset increases. Additionally, the feature extraction process is automatic, whereas other machine learning models require manual configuration.

Deep learning models excel in processing large datasets and approximating continuous relationships within the data, making them highly suitable for handling single-cell data to infer functional GRNs. A notable application is the use of autoencoders for dimension reduction and identifying potential regulatory relationships from various types of single-cell omics input data (Liu et al., 2023a). Additionally, many innovative approaches have emerged to utilize the matched scRNA-seq and scATAC-seq data (Ma et al., 2023a; Yuan and Duren, 2024). For example, Song and colleagues have introduced the multi-task-based MTLRank framework, which incorporates RNA velocity and scATAC-seq to obtain more accurate tissue-specific regulatory networks (Song et al., 2023). However, the application of these novel methods remains limited in plant species (Guo et al., 2024).

While deep learning models demonstrate their flexibility and ability to capture complex patterns, they often require large training datasets and substantial computational resources due to the vast number of parameters involved. Moreover, the models can be less interpretable than traditional models (Ma and Xu, 2022).

It is noteworthy that each model has its pros and cons (Marbach et al., 2012a). For example, correlation coefficient methods are more reliable for loop connections, whereas regression methods are suitable for linear regulatory relationships. Thus, the combination of multiple methods is expected to outperform individual methods (Vignes et al., 2011; Slawek and Arodz, 2013; Zhong et al., 2014).

### 3.3 Integrative GRN construction

In line with the concept of combining different methods in predicting functional GRN, combining physical and functional interactions datasets is also essential to construct comprehensive and high-confidence regulatory networks. The integration process can be achieved by simply using the ChIP-seq data for a TF along with the matched RNA-seq data in the mutant or by employing more advanced algorithms to merge the information from various multi-omics datasets.

#### 3.3.1 Innovative approaches for aggregating TF-binding and gene expression datasets

The process of identifying direct and functional targets of a TF can be achieved by intersecting the TF-binding derived targets with differentially expressed genes identified from perturbations such as overexpression or knock-out of the TF. This method is considered state-of-the-art in TF target identification. However, it is important to note that these two evidence sources rarely converge on a common set of target genes. Despite being widely used as the gold standard, even the bound and differentially expressed genes may not be the validated functional targets (Kang et al., 2020).

To improve the prediction performance of TF-target relationships, a few advanced strategies have been proposed. Kang and coworkers introduced a method called Dual Threshold Optimization (DTO). This method improves the accuracy of identifying direct functional targets by combining data from TF binding sites and TF perturbation responses. The DTO method enhances the convergence of two data types by optimizing the significance thresholds for binding and responsive data (Kang et al., 2020). Morin and colleagues built upon two existing strategies (Tang et al., 2011; Wang et al., 2013) to create a framework to identify and rank TF-target interactions, and identified potential orthologous interactions between humans and mice. This workflow can be scaled to other TFs and offered experimental-level gene summaries evaluated against independent literature evidence (Morin et al., 2023).

#### 3.3.2 Machine-learning based integration framework

To integrate more layers of physical and functional input data, more sophisticated machine-learning methods have been developed for integrative network inference (Mahood et al., 2020). The machine-learning-based methods can be grouped as unsupervised and supervised approaches.

The supervised approach utilizes a regression classifier, which is trained on known regulatory interactions to predict whether an edge (regulatory interaction) exists between TFs and target genes. In contrast, the unsupervised method averages the evidence across different feature-specific networks to generate a comprehensive regulatory network without requiring prior knowledge of regulatory interactions (Marbach et al., 2012b). Both the supervised and unsupervised integrative networks show high coverage. Recently, De Clercq and coworkers applied a supervised learning approach to integrate information about TF-binding, chromatin accessibility, and expression-based regulatory interactions in *Arabidopsis*. The resulting integrated GRN demonstrated high predictive power, facilitating the discovery of previously unidentified regulators (De Clercq et al., 2021).

## 4 Evaluation and downstream analyses of GRNs

After constructing a GRN, evaluating its accuracy and coverage is an essential task. And subsequent downstream analyses can be conducted to extract more biological insights.

### 4.1 Network evaluation

One should bear in mind that the connections in a GRN are hypothetical and require vigorous evaluation of their accuracy. Several common practices have been established to evaluate the biological relevance of the inferred connections (**Li et al., 2015**).

The most common evaluation method involves comparing the inferred GRN with a **“**gold standard**”** network, which is often derived from experimentally verified results, such as loss and gain of function experiments. These wet-lab approaches generate confident regulatory connections by observing the impact of a regulator**’**s expression changes on its target gene (**Kim et al., 2023**). When performing the comparison, one may calculate the average accuracy according to the detection ratio of the verified edges, which is probably flawed due to the sparsity of GRNs. In other words, an algorithm that always predicts the absence of edges could incorrectly achieve high accuracy. Thus, a better approach is to assess the proportion of correctly identified positives relative to all positives (sensitivity or recall) and the proportion of correctly identified positives out of all identified positives (precision or positive predictive value) (Huynh-Thu and Sanguinetti, 2019).

When experimentally validated “gold standard” or any well-accepted high-confidence networks are unavailable, alternative approaches for evaluating gene networks may include cross-validation tests and functional coherent module assessment. Cross-validation tests the accuracy of the reconstructed network by predicting gene functions based on the known functions of network neighbors. Additionally, high-quality networks are expected to exhibit coherent modules of interacting and co-regulated genes. The functional coherence of these modules can be evaluated through enrichment tests of gene function and probabilistic models to predict gene expression within the module (Li et al., 2015).

### 4.2 Downstream analysis

In addition to connecting TFs and their target genes, GRNs can provide further insights into gene functions and associated biological processes through downstream analysis.

#### 4.2.1 Network topological analysis

GRNs often consist of large number of nodes and connections, which renders direct interpretation. Topological analysis has emerged as a useful method for examining the structural properties of these networks, such as node degree distribution, clustering coefficients, and community structures, to detect important patterns and anomalies within the network. In addition to uncovering the underlying structure of the graph, network topological analysis can also assist in identifying influential nodes or edges within the network. For instance, node centrality measures like degree centrality, betweenness centrality, and eigenvector centrality can highlight the most critical nodes in terms of their connectivity and impact on the network. Modularity is another important property of GRN (Segal et al., 2003). Genes within the same module are often co-regulated and often share biological functions. Module detection helps identify sets of genes associated with specific biological processes. For example, Tu and colleagues partitioned a GRN of maize leaves into seven modules. Subsequent analyses using GO terms and MapMan revealed the enrichment of specific functions in each module (Tu et al., 2020).

#### 4.2.2 Comparative gene network analysis

Comparative analysis of GRNs can be used to compare different species, cell types, and treatment conditions. This approach provides more insight than directly comparing sequences or genes (Movahedi et al., 2011; Weston et al., 2011). During interspecific comparisons, it is important to conclusively define gene orthology and to ensure that comparable tissues are being examined.

Previous comparative GRN analysis methods involved pairwise subtraction of TF-gene interactions between GRNs (Thompson et al., 2015; Duren et al., 2021). However, due to the sparse and noisy nature of GRNs, a direct comparison of TF-gene interactions is not good enough. New strategies, such as topic modeling, have been employed to generate dense, low-dimensional representations that filter out the noise in the GRN and more robustly depict the differences in regulatory relationships (Lou et al., 2020).

#### 4.2.3 Prioritizing functional candidate regulators

Pinpointing the key regulatory TF in a network is of great interest in GRN downstream analyses. One approach to identifying these key TFs is to infer TF activities in a specific context using enrichment methods. These methods integrate gene expression with the topological information of GRNs, thereby extracting insights regarding the roles of TFs in particular biological contexts.

Commonly used methods for enrichment analysis include Gene Set Enrichment Analysis (GSEA) and Analysis of Upstream Regulators (AUCell) (Subramanian et al., 2005; Van de Sande et al., 2020). These techniques allow for a thorough analysis that integrates gene expression patterns with the structures of connections. For instance, Yuan and colleagues have utilized the AUCell enrichment method to discover high-activity TFs for each distinct cell type in a maize endosperm single-cell RNA-seq study (Yuan et al., 2024).

Moreover, the application of more sophisticated machine learning models has further advanced the prioritization of TFs. With the known-function genes as training data, these models are capable of identifying the TFs most significantly associated with specific biological processes. For example, NeuralNet algorithm was used to prioritize tassel branch number-related candidate genes (Wang et al., 2023b). Han and coworkers used a similar approach to generate a prediction model based on an integrative map, and predicted which genes are associated with the flowering time pathway (Han et al., 2023).

## 5 Recent advances in regulatory network studies of crop species

In network-related literatures, some focus on developing new inference methods or serving as database resources (**Table 1**); others are dedicated to solving specific biological questions. Many studies with advanced concepts have been conducted in the model plant *Arabidopsis* (**Table 2**).

**Table 1:** Database resources of regulatory network from the past decade.

Species	Database Name	References
Multiple species	ATTED-II (v11)	(Obayashi et al., 2022)
Multiple species	ATTED-II	(Obayashi et al., 2018)
Multiple species	PlantTFDB (v3.0)	(Jin et al., 2014)
Multiple species	PlantTFDB (v4.0)	(Jin et al., 2017)
Multiple species	PlantRegMap	(Tian et al., 2020)
Multiple species	PlantPAN (v2.0)	(Chow et al., 2016)
Multiple species	PlantPAN (v3.0)	(Chow et al., 2019)
Multiple species	PlantPAN (v4.0)	(Chow et al., 2024)
Multiple species	ChIP-Hub	(Fu et al., 2022)
Multiple species	KnockTF	(Feng et al., 2024)
Multiple species	Plant-DTI	(Ruengsrichaiya et al., 2022)
Multiple species	PlaPPISite	(Yang et al., 2020)
*Arabidopsis*/Animal species	UniBind	(Puig et al., 2021)
*Arabidopsis*/Maize/Rice	ConnecTF	(Brooks et al., 2021)
Soybean	SoyNet	(Kim et al., 2017a)
Tomato	TomatoNet	(Kim et al., 2017b)
*Arabidopsis*	TF2Network	(Kulkarni et al., 2018)
*Arabidopsis*	Cistrome	(O'Malley et al., 2016)
*Arabidopsis*	AraNet (v2)	(Lee et al., 2015b)
*Arabidopsis*	AraPPINet	(Zhao et al., 2019)
*Arabidopsis*	AGRIS	(Yilmaz et al., 2011)
Rice	RicePPINet	(Liu et al., 2017)
Rice	RiceENCODE	(Xie et al., 2021)
Rice	NetREx	(Sircar et al., 2022)
Rice	RiceTFtarget	(Zhang et al., 2023a)
Rice	RiceNet (v2)	(Lee et al., 2015a)
Maize	MaizeNetome	(Feng et al., 2023)
Maize	CORNET (v2.0)	(De Bodt et al., 2012)
Maize	MaizeNet	(Lee et al., 2019)
Wheat	WheatNet	(Lee et al., 2017b)
Wheat	WheatOmics	(Ma et al., 2021)
Wheat	wGRN	(Chen et al., 2023b)
Wheat	Wheat-RegNet	(Tang et al., 2023)
Wheat	WheatCENet	(Li et al., 2023b)

**Table 2:** Selected network-related studies in *Arabidopsis* from the past decade.

Species	Research Objective	Brief Description	References
*Arabidopsis*	Methodology	MICRAT	(Yang et al., 2018)
*Arabidopsis*	Biology question	PPI network	(Nietzsche et al., 2016)
*Arabidopsis*	Biology question	GRN of root	(Santuari et al., 2016)
*Arabidopsis*	Biology question	GRN of root stem cell	(de Luis Balaguer et al., 2017)
*Arabidopsis*	Biology question	JA signal pathway (GRN)	(Hickman et al., 2017)
*Arabidopsis*	Methodology	CoReg package	(Song and Li, 2023)
*Arabidopsis*	Biology question	Metabolic Pathways	(Wisecaver et al., 2017)
*Arabidopsis*	Biology question	Flower development (GRN)	(Chen et al., 2018)
*Arabidopsis*	Methodology	Protein binding microarrays (PBM)	(Franco-Zorrilla et al., 2014)
*Arabidopsis*	Biology question	Nitrogen metabolism	(Gaudinier et al., 2018)
*Arabidopsis*	Methodology	Chromatin accessibility	(Sullivan et al., 2014)
*Arabidopsis*	Biology question	Single and combined stresses	(Barah et al., 2016)
*Arabidopsis*	Biology question	Nitrogen signaling	(Varala et al., 2018)
*Arabidopsis*	Biology question	Response to elevated CO_2_	(Cassan et al., 2023)
*Arabidopsis*	Biology question	NLP7 regulon	(Alvarez et al., 2020)
*Arabidopsis*	Biology question	Nitrogen signaling	(Brooks et al., 2019)
*Arabidopsis*	Methodology	EXPLICIT	(Geng et al., 2021)
*Arabidopsis*	Methodology	EXPLICIT-Kinase	(Peng et al., 2022)
*Arabidopsis*/Rice/Maize	Methodology	TSPTFBS 2.0	(Cheng et al., 2023)
*Arabidopsis*	Methodology	ConSReg	(Song et al., 2019)
*Arabidopsis*/Rice	Methodology	Comparative analysis of GRN	(Wang et al., 2020b)
*Arabidopsis*	Biology question	Response to JA	(Zander et al., 2020)
*Arabidopsis*	Biology question	ROS signaling	(De Clercq et al., 2021)
*Arabidopsis*	Methodology	Annotation of unknown gene	(Depuydt and Vandepoele, 2021)
*Arabidopsis*	Biology question	scATAC-seq of root	(Dorrity et al., 2021)
*Arabidopsis*/Rice/Maize	Methodology	MINI-EX	(Ferrari et al., 2022)
*Arabidopsis*/Rice/Maize /Tomato	Methodology	MINI-EX V2.0	(Jasper et al., 2023)
*Arabidopsis*/maize	Methodology	MINI-AC	(Manosalva Perez et al., 2024)
*Arabidopsis*	Biology question	scRNA-seq of BR root	(Nolan et al., 2023)
*Arabidopsis*/Maize	Biology question	Nitrogen responsive GRN	(Cheng et al., 2021)

For example, Hickman and colleagues conducted a time-series experiment to study the regulation of JA response in *Arabidopsis*. They used RNA-seq data from 14-time points on MeJA (methylated ester of JA) treated leaf and constructed a dynamic model of the JA GRN. This study offers significant advances in our understanding of how plants dynamically regulate the JA signaling pathway in response to environmental cues and lays an foundation for further investigating the complex transcriptional programs underlying plant stress responses and developmental processes (Hickman et al., 2017). Zender and coworkers combined time-series transcriptome, proteome, and phosphoproteome data to reconstruct GRNs, predict new components involved in the JA signaling pathway, and validate these new genes through genetic mutants. This work demonstrates the power of integrative multi-omics approach to provide fundamental biological insights into plant hormone responses (Zander et al., 2020). De Clercq and colleagues have combined networks based on DNA motifs, open chromatin, transcription factor (TF) binding, and expression-based interactions through a supervised learning approach. The integrated GRN outperforms the individual input networks in predicting known regulatory interactions. They also experimentally validated many TFs involved in reactive oxygen species (ROS) stress regulation, including 13 novel ROS regulators (De Clercq et al., 2021).

Researchers also construct many regulatory networks in crop species, particularly in cereals such as maize and wheat. We have endeavored to summarize these works with a focus on presenting the cutting-edge findings rather than aiming for comprehensiveness. We highlight a selection of literatures from the last decade (**Table 3**).

**Table 3:** Selected network-related studies in crops from the past decade.

Species	Research Objective	Descriptions	References
Rice	Biology question	Pollen development	(Lin et al., 2017)
Rice	Biology question	Response to Cadmium stress	(Wang et al., 2020a)
Rice	Methodology	Improvement of PBM	(Kim et al., 2021a)
Rice	Biology question	Gene editing promoter of *IPA1*	(Song et al., 2022)
Rice	Biology question	Phosphate starvation response	(Shi et al., 2021)
Rice	Biology question	Biotic stress response	(Lee et al., 2011)
Rice	Biology question	scRNA-seq GRN	(Xie et al., 2020)
Rice	Biology question	Agronomic traits	(Zhang et al., 2022a)
Rice	Biology question	Low temperature response	(Wang et al., 2022a)
Maize	Biology question	Leaf development	(Yu et al., 2015)
Maize	Biology question	Leaf development	(Tu et al., 2020)
Maize	Biology question	Seed development	(Zhan et al., 2015)
Maize	Biology question	Seed development	(Liu et al., 2016)
Maize	Biology question	Seed development	(Yi et al., 2019)
Maize	Biology question	Seed development	(Xiong et al., 2017)
Maize	Biology question	Seed development	(He et al., 2024)
Maize	Biology question	Inositol phosphate metabolism	(Zhang et al., 2016b)
Maize	Biology question	Developmental atlas	(Walley et al., 2016)
Maize	Biology question	Gene network of grey leaf spot	(Christie et al., 2017)
Maize	Methodology	Optimize GCN construction	(Huang et al., 2017)
Maize	Biology question	Phenolic compound biosynthesis	(Yang et al., 2017)
Maize	Biology question	TFBS of ARF family	(Galli et al., 2018)
Maize	Biology question	Tissue-specific GRN	(Huang et al., 2018)
Maize	Biology question	Inflorescence development	(Parvathaneni et al., 2020)
Maize	Biology question	Meta GRNs using RNA-seq data	(Zhou et al., 2020)
Maize	Biology question	scATAC-seq of 6 tissues	(Marand et al., 2021)
Maize	Biology question	scRNA-seq of ear	(Xu et al., 2021)
Maize	Biology question	scRNA-seq of leaf	(Tao et al., 2022)
Maize	Biology question	Spatial transcriptomics of seed	(Fu et al., 2023)
Maize	Biology question	scRNA-seq of endosperm	(Yuan et al., 2024)
Maize	Biology question	Multi-omics integrative network	(Han et al., 2023)
Maize	Biology question	Early shade avoidance response	(Wang et al., 2016)
Maize	Biology question	Translatome-transcriptome GRN	(Zhu et al., 2023b)
Maize	Biology question	Prioritizing Metabolic GRN	(Gomez-Cano et al., 2024)
Maize	Biology question	Lipid metabolism	(de Abreu et al., 2018)
Maize	Biology question	UV-B stress response	(Gupta et al., 2018)
Maize	Biology question	Seed dormancy	(Ma et al., 2023b)
Maize	Biology question	Development, nutrients utilization, metabolism, and stress response	(Ma et al., 2017)
Maize	Biology question	Responses to Puccinia sorghi	(Kim et al., 2021b)
Maize	Biology question	Bundle sheath and mesophyll cells network	(Dai et al., 2022)
Maize	Biology question	WGCNA of bundle sheath and mesophyll cells	(Tao and Zhang, 2022)
Maize	Methodology	ChIA-PET	(Peng et al., 2019)
Wheat	Biology question	Grain transcriptome	(Pfeifer et al., 2014)
Wheat	Biology question	The transcriptional landscape of polyploid wheat	(Ramírez-González et al., 2018)
Wheat	Biology question	Regulating Senescence	(Borrill et al., 2019)
Wheat	Biology question	Fusarium head blight resistance	(Sari et al., 2019)
Wheat	Biology question	Embryogenesis and grain development	(Xiang et al., 2019)
Wheat	Biology question	Chloroplast biogenesis	(Loudya et al., 2021)
Wheat	Biology question	Evolutionary rewiring of the wheat transcriptional regulatory network	(Zhang et al., 2021)
Wheat	Biology question	Construction of GRN with DAP-seq data	(Zhang et al., 2022b)
Wheat	Biology question	Spike architecture	(Wang et al., 2017)
Wheat	Biology question	Biologically-Relevant	(Harrington et al., 2020)
Wheat	Biology question	Integrate gene regulatory network and genetic variation	(Ai et al., 2024)
Wheat	Biology question	Spike development	(Lin et al., 2024)
Wheat	Biology question	Regeneration	(Liu et al., 2023b)
Wheat	Biology question	scRNA-seq of root	(Zhang et al., 2023b)
Sorghum	Biology question	Bioenergy stems	(Fu et al., 2024)
Cotton	Biology question	Oil accumulation	(Ma et al., 2024)
Cotton	Biology question	Low light intensity	(Zhao et al., 2024)
Cotton	Biology question	Seed yield	(Zhao et al., 2023)
Rapeseed	Biology question	Oleic acid content	(Yao et al., 2020)
Soybean	Biology question	Salt stress	(Yang et al., 2019)
Tomato	Biology question	Drought-responsive	(Chowdhury et al., 2023)
Sweet potato	Biology question	Chlorogenic acid biosynthesis	(Xu et al., 2022a)
Banana	Biology question	Fruit ripening	(Kuang et al., 2021)
Multiple species	Methodology	cisDynet software	(Zhu et al., 2023a)

### 5.1 GCN studies in crops

Early-stage studies often relied solely on bulk gene expression data, typically obtained from specific plant tissues or organs, to construct functional GCNs.

For example, Pfeifer and colleagues analyzed gene expression in developing wheat grains and constructed a co-expression network comprising 25 modules. These modules displayed unique spatiotemporal characteristics that can be distinguished based on grain cell types or developmental stages (Pfeifer et al., 2014). To provide insights into the coordination of individual homoeologs underlying various traits in wheat, the coexpression networks were constructed from nonstress tissue-specific and stress-related RNA-seq samples. These networks highlight the extensive coordination of homoeologs throughout development and in response to various stresses and offer a platform to identify candidate genes for agronomic traits (Ramírez-González et al., 2018). Huang and coworkers evaluated various parameters for data normalization and different inference methods for constructing a large GCN in maize using RNA-Seq data. The analysis revealed that increasing sample size positively impacts network performance, emphasizing the importance of sample size for the construction of accurate GCNs (Huang et al., 2017). To extend the knowledge of salt response in soybean, Hu and colleagues clustered differentially expressed genes between a salt-tolerant and a salt-hypersensitive cultivar. They constructed undirected networks representing their co-expression patterns based on Pearson’s correlation coefficients. The network analysis unveiled several candidate pathways critical in salt responses, including phytohormone signaling, oxidoreduction, phenylpropanoid biosynthesis, and others (Hu et al., 2022).

While GCNs cannot directly define the regulatory relationships between TFs and their downstream targets, the connectivity results are widely used to refine the findings from genome-wide association studies (GWAS) and quantitative trait locus (QTL) analyses. This integration enables the effective prediction of novel candidate genes. For example, a weighted GCN analysis was used to identify connected genes associated with Fusarium head blight (FHB) resistance and pinpointed candidate hub genes within the interval of three previously reported FHB resistance QTL in wheat (Sari et al., 2019). Yao and coworkers combined GWAS and co-expression network analyses to uncover candidate genes involved in the accumulation of oleic acid content in rapeseed (Yao et al., 2020). GCN analysis and genome-wide association studies (GWAS) were combined to elucidate the regulatory pathways and identify candidate genes responsible for pre-harvest sprouting and seed dormancy traits in maize (Ma et al., 2023b).

### 5.2 Networks based on TF-DNA binding in crops

Establishing direct physical interactions between TF and DNA has been a major research focus. In addition to TFBSs obtained from experimental techniques such as ChIP-seq, many TFBSs have been predicted by computational algorithms. For example, Yu et al. collected transcriptomics data from developing maize leaves and used co-expression data along with enrichment analysis to predict overrepresented motifs in the promoter sequences and the potential TFBSs of key TFs (Yu et al., 2015).

A few databases and newly developed inference methods have significantly expanded the available information on TF-DNA binding interactions. PlantPAN, which has collected a comprehensive set of public ChIP-seq datasets, is a valuable resource for plant TF-TFBS interactions. It offers the most complete plant PWMs for analyzing TFBSs and effective tools for predicting TFBSs in conserved regions of a given promoter. The latest version, PlantPAN 4.0, provides a non-redundant set of 3,428 matrices for 18,305 TFs of 115 plant species (Chow et al., 2024). Another valuable resource is ChIP-Hub, a comprehensive and standardized platform for exploring the regulome of plants. It collects over 10,000 datasets from 41 plant species and processes them based on ENCODE standards. As an application example, an extensive survey was performed to examine the co-associations among various regulators, enabling the construction of a hierarchical regulatory network spanning a broad developmental context (Fu et al., 2022).

Meanwhile, wet-lab approaches persist in being actively employed to extend experimental TF-DNA binding in plants. In wheat, Zhang and colleagues have successfully obtained high-quality DNA binding profiles for 53 environmentally responsive TFs using DAP-seq. Interestingly, the study found that 85% of the *in vitro* identified TFBSs were located within transposable elements and associated with regulatory sequences specific to the wheat lineage (Zhang et al., 2021). In a subsequent study by the same group, genomic binding profiles were generated for a larger set of TFs, enabling the assembly of a wheat GRN encompassing connections among 189 TFs and 3,714,431 regulatory elements (Zhang et al., 2022b). These results provide valuable insights into the transcriptional regulatory mechanisms in wheat. Several remarkable advances were also made in maize. Ricci and coworkers performed DAP-seq on 32 TFs, indicating that the distal accessible chromatin regions were enriched for TFBS (Ricci et al., 2019). Additionally, interaction maps were generated for 14 maize TFs from the ARF family, revealing both specific and redundant binding events of ARF TFs (Galli et al., 2018). Furthermore, 104 maize TFBS datasets were yield by ChIP-seq with transient expressed proteins to construct the leaf regulatory network (Tu et al., 2020).

### 5.3 Inference of GRN using expression data in crops

GRNs derived from gene expression profiles are not limited by the availability of TF-DNA binding data and are widely used in various biological contexts. A GRN was inferred by modeling 78 maize seed transcriptome to identify key genes involved in seed development. The network analysis unraveled highly interwoven communities and identified key genes and regulatory modules associated with nutrient transport and imprinting patterns, which are crucial for maize seed development (Xiong et al., 2017). Utilizing the GENIE3 software package with a number of RNA-Seq data, Huang and colleagues constructed four tissue-specific GRNs in maize. They further predicted key TFs for each specific tissue (Huang et al., 2018).

Zhou and colleagues present a standardized pipeline using machine learning algorithms along with transcriptomic data to predict GRNs (Zhou et al., 2020). They analyzed a large collection of transcriptome datasets, resulting in 45 GRNs. The networks exhibited significant enrichment for biologically relevant interactions, with each GRN capturing diverse biological processes. This uniform pipeline can be applied to other species with available expression data (Zhou et al., 2020). To comprehensively elucidate the chloroplast biogenesis process, Loudya and colleagues present a biologically informed GRN. The network prediction suggests that the regulators of chloroplast genes are differentially involved across various leaf developmental stages in wheat (Loudya et al., 2021).

Similar to GCN, GRN can also be combined with QTL and GWAS results to predict candidate genes for specific traits. For example, Zhao and colleagues designed an integrative analysis combining eQTL, GWAS, and GRN to characterize the genetic basis of cotton yield. Several high-ranking causal genes identified from the GRN were validated for their functional impacts on cotton seed development (Zhao et al., 2023).

### 5.4 Integrative network construction with multi-omics data in crops

Genomics and functional genomics studies on rice have been at the forefront among crop plants. Rice also serves as a leading model in integration network studies. The RiceNet (v2) web resource, launched in 2014, provides an integrative network for rice. This network combined co-functional links based on genomic context similarity, connections inferred from co-expression patterns, and protein-protein interactions. Its utility in prioritizing candidate genes involved in rice biotic stress responses has been demonstrated (Lee et al., 2015a). Another significant pioneering study created a comprehensive developmental atlas of maize with multi-omics data. Integrative GRNs were constructed based on mRNA, protein, and phosphor-protein data, resulting in improved predictive power. This work enhanced our understanding of the complex regulatory mechanisms in maize (Walley et al., 2016).

The integration of multi-omics data has become increasingly prevalent in studies using network-based approaches. Han and colleagues have successfully constructed a large-scale PPI network in maize. An integrated map was constructed incorporating data from four different layers: three-dimensional genomics, transcriptomics, proteomics, and protein-protein interactions. Leveraging this multi-omics network and machine learning-based prediction approaches, novel candidate key genes involved in various regulatory pathways, such as flowering time, have been predicted and genetically validated (Han et al., 2023). Gomez-Cano and coworkers analyzed ~4.6M interactions, including co-expression networks, TF-DNA interaction experiments, and expression quantitative trait loci (eQTL) to construct GRNs and pinpointed key regulators associated with hormone, metabolic, and developmental processes (Gomez-Cano et al., 2024). Additionally, several studies integrate a large amount of omics data, including both physical interactions and functional regulation relationships, in wheat (Chen et al., 2023; Tang et al., 2023). Similar integrated analysis has also been conducted in cotton (Zhao et al., 2024).

The integration of network and genetic mapping data, such as GWAS, further enhances the predictive power for identifying significant genes. Lin and colleagues thoroughly examined the transcriptome and epigenome profiles of the developing spike in an elite wheat cultivar. Through the integration of regulatory networks with GWAS, key genes affecting the spike architecture were pinpointed (Lin et al., 2024).

### 5.5 Regulatory networks at a high spatial resolution in crops

Regulatory networks relying on bulk data have several limitations. These models typically only capture generalized connection patterns, which obscure distinct regulatory interactions unique to certain cell types. Furthermore, bulk data often fails to differentiate the cellular states, which can significantly impact gene regulation. In contrast, approaches such as microdissection and single-cell technologies enable the discovery of regulatory networks at greater spatial resolution.

Zhan and coworkers used laser-capture microdissection with RNA-Seq to profile gene expression in each dissected cell compartment of the maize kernel (Zhan et al., 2015). They constructed an unbiased GCN and detected sub-network modules containing genes predominantly expressed in a single compartment or ubiquitously expressed across multiple compartments. These results offer a high-resolution gene expression atlas of maize kernel and contribute to uncovering regulatory interactions associated with the differentiation of major endosperm cell types (Zhan et al., 2015).

With the advent of sing-cell omics, Marand and colleagues generated a cis-regulatory atlas using single-cell ATAC-seq in maize. They profiled over 72,000 nuclei across six maize organs and identified TFs coordinated with chromatin interactions by analyzing patterns of co-accessible CREs. This comprehensive cis-regulatory atlas at single-cell resolution is a valuable resource to study the gene regulation in maize (Marand et al., 2021). The researchers from Vandepoele’s group have developed computational methods named MINI-EX and MINI-AC to explore cell type-specific regulatory interactions. MINI-EX utilizes expression-based GRNs derived from single-cell RNA-seq data and TF binding motifs to predict cell type-specific regulons. On the other hand, MINI-AC combines accessible chromatin (AC) data from either bulk or single-cell experiments with TF binding motifs to construct GRNs (Ferrari et al., 2022; Manosalva Perez et al., 2024). The application of MINI-EX has successfully identified regulons (groups of genes co-regulated by a shared TF) across major cell types in *Arabidopsis*, rice, and maize. Moreover, this method effectively prioritized established key regulons based on their network characteristics, such as connectivity and centrality, and unveiled several previously unidentified transcriptional regulators (Ferrari et al., 2022). Similarly, MINI-AC has also demonstrated superior performance compared to other techniques in accurately identifying TFBS. Maize has a complex genome and abundant distal AC regions. MINI-AC successfully inferred leaf GRNs containing experimentally confirmed interactions between TFs and target genes from both proximal and distal regions in maize. It is also a robust tool for pinpointing both known and novel candidate regulators (Manosalva Perez et al., 2024).

Recently, Yuan and coworkers focused on the differentiation stage of maize endosperm. They performed single-cell RNA-seq combined with TFBS profiling using ampDAP-seq to construct a high-confidence GRN and identified key regulators in five distinct cell types (Yuan et al., 2024). Fu and colleagues utilized the endosperm spatial transcriptome data during the grain-filling stage. They successfully predicted and identified the function of the candidate sucrose transporter genes (*SUTs*) in endosperm transfer cells facilitated by GCN analysis (Fu et al., 2023).

### 5.6 Utilizing clues from networks to answer biological questions in crops

Unlike traditional forward genetics, a new research paradigm is emerging for gene function studies, wherein candidate genes are determined through hints from networks. Y1H is an easy approach used to identify the direct binding between TFs and the promoters of their targets. These direct regulatory relationships have great value in guiding the selection of key regulators for functional characterization.

For instance, Gaudinier and colleagues used enhanced Y1H assays to screen for *Arabidopsis* TFs binding to the promoters of genes associated with nitrogen metabolism and signaling, resulting in a network comprising 1,660 interactions. This network unveiled a hierarchical regulation of these TFs. Mutants of 17 prioritized key TFs exhibited significant alterations in at least one root architecture trait. The identification of regulatory TFs in the nitrogen-regulatory framework holds promise for enhancing agricultural productivity (Gaudinier et al., 2018). Similarly, Shi and coworkers uncovered TFs that regulate genes related to mycorrhizal symbiosis using Y1H. They screened more than 1500 rice TFs for binding to 51 selected promoters, and constructed a highly interconnected network. Interestingly, many of the TF in this network are involved in the conserved P-sensing pathway. With functional analyses of selected genes, this study elucidates the extensive regulation of mycorrhizal symbiosis by both endogenous and exogenous signals (Shi et al., 2021).

Ji and colleagues constructed a co-expression network to identify regulatory factors during the grain-filling stage of maize endosperm, and identified hundreds of candidate TFs using 32 storage reserve-related genes as guides. In addition to known regulators of storage proteins and starch, the study uncovered novel TFs, such as *GRAS11*, involved in endosperm development. They further characterized the function of *GRAS11* through detailed functional analysis (Ji et al., 2022). High-temporal-resolution RNA sequencing was conducted on the basal and upper regions of maize kernels. Weighted gene co-expression network analyses were performed, identifying numerous hub regulators that are worthy of subsequent functional characterization (He et al., 2024).

Collectively, these studies have provided significant insights into transcriptional regulation programs and rich data resources. GCN analyses have identified modules and candidate genes associated with various traits. Experimental determination of TFBSs, aided by computational predictions, has enabled the construction of regulatory networks, revealing novel regulators. Integration of multi-omics data has improved the predictive power of GRNs. High-resolution spatial techniques have uncovered cell-type-specific regulatory interactions, providing a more nuanced understanding of gene regulation. Overall, the advancements in regulatory network studies of crop species have substantially enhanced our understanding of the complex transcriptional programs governing plant growth, development, and responses to biotic and abiotic stresses.

## 6 Challenges and future perspectives

The precise manipulation of gene expression can be used to breed crops with desirable traits. The inference and analysis of regulatory networks will assist in crop improvement efforts. Despite the significant breakthroughs in regulatory network studies in recent years, there is still potential for enhancing the confidence of the inferred interactions.

### 6.1 Network validation is a complicated task

The validation of regulatory networks is crucial to ensure that these networks accurately reflect the biological processes of interest. GRN evaluation commonly requires a thorough comparison of predicted interactions with the “gold standard” derived from wet-lab experiments, such as independently generated TF-DNA binding data and perturbation tests on the regulator TFs, as discussed in **Section 4.1**. However, the experimental “gold standard” is often unavailable or inadequate. Noteworthy, even if TF binding to a target is confirmed by *in vivo* ChIP assay, it does not necessarily imply that this TF can activate or repress the target gene. An alternative approach to test whether a TF regulates a particular gene is to perturb the TF expression and check how this perturbation affects the expression of target genes. While this approach shows promise due to its inherent causality, perturbation experiments are time consuming and costly. Noteworthy, they are likely not to work well as hindered by the widely existed compensatory mechanisms in crop plants. Due to different validation methods have their own limitations, utilizing diverse assessment strategies to evaluate a given GRN may be a smart way.

### 6.2 Increasing spatiotemporal resolution of networks

Regulation of gene expression is a dynamic process. High-temporal resolution studies have revealed fluctuations in gene expression levels in maize kernels within a small time window (Yi et al., 2019). From the perspective of network inference, there may be lack of expression correlation between target genes and their regulatory TFs because of the temporal lag between TF binding and the accumulation of mRNA transcripts. Thus, improving the spatiotemporal resolution of gene expression profiles and TF-DNA binding data is imperative for network construction.

Although new technologies have been developed to more efficiently acquire multi-omics data from a single plant cell (Xu et al., 2022b), the primary challenge arises from technical difficulties in the preparation of high-quality protoplasts in plants. Recently, to address challenges in protoplasting experiments, several optimized enzymatic cell wall digestion protocols have been developed for various species (Ye et al., 2022; Wang et al., 2022b; Chen et al., 2023a). Wang and colleagues introduced a new method that involves two consecutive digestion processes with different enzymatic buffers, significantly enhancing the efficiency and viability of protoplast preparation across diverse plant tissues (Wang et al., 2022b). However, the conversion of cells into protoplasts is still not feasible for many types of plant tissues. Alternatively, recent single-nucleus techniques offer broader applicability across different tissues. Nevertheless, it’s important to note that nuclear RNA and cytoplasmic RNA should not be considered equivalent.

### 6.3 Hurdles in linking GRN to agronomic traits

There is still a large gap between the knowledge derived from GRNs and their manifestation in agronomic traits. Firstly, GRNs involve a complex interplay among thousands of genes and TFs that underlie various biological processes. Deciphering how perturbations in these regulatory networks impact gene expression remains a challenge. Secondly, the relationship between gene expression levels and traits is nonlinear and polygenic. Therefore, predicting observable traits from changes in gene expression, especially considering the influence of environmental factors, is difficult.

At the current stage, it is feasible to modulate specific metabolic pathways based on network information. The activity of one or a few TFs can regulate multiple steps of metabolic pathways. Thus, manipulating the expression of TFs probably has a greater impact on metabolism pathways than modifying cis-regulatory elements of enzyme-coding genes. For example, flavonoids are considered valuable compounds in plant metabolic engineering. Increasing flavonoid levels can be achieved by manipulating their transcription regulatory elements, resulting in the development of plants with high anthocyanin content (Jiang et al., 2023). For the enhancement of oil production, *WRINKLED1* is a conserved transcription factor involved in the regulation of fatty acid biosynthesis in diverse angiosperms. Transgenic plants that overexpress the *WRINKLED1* gene show promising outcomes in increasing the oil content of maize, soybean, and rice. As *WRINKLED1* also modulates targets that affect plant growth and development. It is important to consider the shared regulatory network when utilizing it to engineer plant oil production (Yang et al., 2022).

However, achieving the modulation of complex traits, such as yield and quality, which are determined by multiple factors, remains challenging. The regulatory mechanisms that directly impact these processes have not been thoroughly characterized. And these complex traits are often influenced by environmental factors and are sensitive to the interplay between genotype and environment.

### 6.4 Future directions

In response to current challenges, there are several aspects in future network-related research that need to be strengthened. Firstly, integrating more omics data can enhance the predictive power of networks by merging diverse complementary information. Secondly, improving spatiotemporal resolution relies on the development of more sensitive, convenient, and cost-effective technologies. Lastly, the application of deep learning models, which can better integrate massive amounts of data and extract reliable and useful information from them, provides an opportunity to construct more accurate GRNs.

## Author contributions

QH: Writing – original draft, Writing – review & editing. RS: Writing – review & editing. ZM: Writing – review & editing, Writing – original draft.
